# Supplementary material for: Analysis of microbial diversity and functions in sediments and overlying water of the Shiliu River
Source: PeerJ. 2025 Aug 29;13:e19979. doi: 10.7717/peerj.19979 (PMC12401026; doi:10.7717/peerj.19979)
Supplement: Supplemental Information 10 [file peerj-13-19979-s010.docx]

**Analysis of Microbial Diversity and Functions in Sediments and Overlying Water of Shiliu River**

Yazi Li^1^, Shuhong Zhang^1^, Yumei Guo^2^, Ke Xu^1^, Xiqing Zhang^3^, Mengfei Pan^3^, Qiaoping Sun^4^, Yanfang Zhang^4^, Yongshan Fan^1*^

1. Department of Life Sciences, Hebei Key Laboratory of Plant Biotechnology Research and Application, Tangshan Normal University, Tangshan, Hebei, China.

2. Institute for Microbiological Examination, Shijiazhuang Center for Disease Control and Prevention, Shijiazhuang, Hebei, China.

3. College of Life Sciences, Agricultural University of Hebei, Baoding, Hebei, China.

4. Hebei Green Dream Environmental Science & Technology Co., Ltd, Tangshan, Hebei, China

*Corresponding Author:

Yongshan Fan^1^

Tangshan, Hebei 063000, China

Email address: [lyz19920105@163.com](mailto:lyz19920105@163.com)

**This file includes:**

Supplementary Table 1-4

**Supplementary Table 1** Statistics of the sequencing data processing results of bacterial samples from sediments (B1, B2, B3) and overlying water (D1, D2, D3).

| Group | Sample | SeqNum | BaseNum | MeanLen | MinLen | MaxLen |
| --- | --- | --- | --- | --- | --- | --- |
| B | B1 | 52992 | 22159775 | 418.17 | 352 | 441 |
| B | B2 | 59213 | 24698844 | 417.12 | 350 | 464 |
| B | B3 | 58570 | 24362868 | 415.96 | 353 | 469 |
| D | D1 | 66646 | 27784248 | 416.89 | 350 | 472 |
| D | D2 | 51638 | 21473986 | 415.86 | 352 | 470 |
| D | D3 | 63295 | 26242776 | 414.61 | 350 | 443 |

**Supplementary** Table 2 Statistics of the sequencing data processing results of fungal samples from sediments (B1, B2, B3) and overlying water (D1, D2, D3).

| Group | Sample | SeqNum | BaseNum | MeanLen | MinLen | MaxLen |
| --- | --- | --- | --- | --- | --- | --- |
| B | B1 | 192571 | 45304369 | 235.26 | 105 | 451 |
| B | B2 | 122682 | 28249737 | 230.27 | 105 | 450 |
| B | B3 | 160558 | 37191308 | 231.64 | 102 | 450 |
| D | D1 | 107075 | 26007021 | 242.89 | 108 | 451 |
| D | D2 | 147133 | 34958026 | 237.59 | 102 | 450 |
| D | D3 | 131222 | 29534446 | 225.07 | 105 | 447 |

**Supplementary Table 3** Statistical analysis of the Alpha diversity index of bacteria in sediment (B1, B2, B3) and overlying water (D1, D2, D3) samples

| Sample | Shannon | Chao1 | Ace | Simpson | Shannoneven | Coverage |
| --- | --- | --- | --- | --- | --- | --- |
| B1 | 6.510 | 2828.844 | 2939.549 | 0.005 | 0.827 | 0.987 |
| B2 | 6.600 | 3060.748 | 3188.698 | 0.004 | 0.830 | 0.986 |
| B3 | 6.487 | 2967.011 | 3076.203 | 0.005 | 0.820 | 0.986 |
| D1 | 5.001 | 1239.679 | 1280.152 | 0.015 | 0.712 | 0.996 |
| D2 | 4.738 | 1631.004 | 1754.111 | 0.023 | 0.661 | 0.990 |
| D3 | 4.548 | 1635.618 | 1774.890 | 0.030 | 0.632 | 0.991 |

**Supplementary Table 4** Statistical analysis of the Alpha diversity index of fungal in sediment (B1, B2, B3) and overlying water (D1, D2, D3) samples

| Sample | Shannon | Chao1 | Ace | Simpson | Shannoneven | Coverage |
| --- | --- | --- | --- | --- | --- | --- |
| B1 | 4.731 | 947.439 | 918.602 | 0.026 | 0.699 | 0.999 |
| B2 | 4.821 | 1031.204 | 1037.732 | 0.024 | 0.699 | 0.999 |
| B3 | 4.294 | 930.000 | 928.520 | 0.053 | 0.632 | 0.999 |
| D1 | 4.140 | 533.071 | 637.249 | 0.040 | 0.698 | 0.994 |
| D2 | 3.563 | 572.706 | 565.621 | 0.078 | 0.583 | 0.998 |
| D3 | 2.657 | 243.955 | 242.182 | 0.142 | 0.496 | 0.999 |
